# Diabetes management intervention studies: lessons learned from two studies

**DOI:** 10.1186/s13063-020-05017-3

**Published:** 2021-01-18

**Authors:** Bettina Petersen, Iris Vesper, Bernhild Pachwald, Nicole Dagenbach, Sina Buck, Delia Waldenmaier, Lutz Heinemann

**Affiliations:** 1 Roche Diabetes Care GmbH, Mannheim, Germany; 2 Institut für Diabetes-Technologie, Forschungs- und Entwicklungsgesellschaft mbH an der Universität Ulm, Lise-Meitner-Strasse 8/2, 89081 Ulm, Germany; 3 Science Consulting in Diabetes GmbH, Neuss, Germany

**Keywords:** Diabetes, Study performance, Behavior-based, Medical device studies, Adaptive study design, Study effect, Randomized controlled trials, ProValue, STeP

## Abstract

**Introduction:**

Several clinical studies investigated improvements of patient outcomes due to diabetes management interventions. However, chronic disease management is intricate with complex multifactorial behavior patterns. Such studies thus have to be well designed in order to allocate all observed effects to the defined intervention and to exclude effects of other confounders as well as possible.

This article aims to provide challenges in interpreting diabetes management intervention studies and suggests approaches for optimizing study implementation and for avoiding pitfalls based on current experiences.

**Main body:**

Lessons from the STeP and ProValue studies demonstrated the difficulty in medical device studies that rely on behavioral changes in intervention group patients. To successfully engage patients, priority should be given to health care professionals being engaged, operational support in technical issues being available, and adherence being assessed in detail.

Another difficulty is to avoid contamination of the control group with the intervention; therefore, strict allocation concealment should be maintained. However, randomization and blinding are not always possible. A limited effect size due to improvements regarding clinical endpoints in the control group is often caused by the Hawthorne effect. Improvements in the control group can also be caused with increased attention paid to the subjects. In order to reduce improvements in the control group, it is essential to identify the specific reasons and adjust study procedures accordingly. A pilot phase is indispensable for this. Another option is to include a third study arm to control for enhanced standard of care and study effects. Furthermore, retrospective data collection could be a feasible option. Adaptive study designs might reduce the necessity of a separate pilot study and combine the exploratory and confirmatory stages of an investigation in one single study.

**Conclusion:**

There are several aspects to consider in medical device studies when using interventions that rely on changes in behavior to achieve an effective implementation and significant study results. Improvements in the control group may reduce effect sizes and limit statistical significance; therefore, alternatives to the traditional randomized controlled trials may be considered.

## Introduction

Patients with diabetes require a life-long treatment that is not limited to a standardized intake of drugs, but requires a more complex disease management. In particular, type 1 diabetes management involves frequent treatment decisions like adjustment of insulin doses depending on the current glucose status, meal intake, and physical activity level. This requires the use of medical devices, adequate handling by the patient, and translation of the measurement results into appropriate therapeutic decisions. Health care professionals (HCPs) support patients with regular monitoring of markers of glucose control and adjustment of the treatment plan. These factors have a complicated interaction with one another to influence the achievement of a therapeutic goal. If individual components of diabetes management are investigated, e.g., in a clinical study, this interaction has to be taken into account. For example, frequent use of a CGM system and adequate interpretation of glucose values will more likely lead to improvements in diabetes management [1].

Therapeutic improvements that are observed as a result of device usage are not driven by the device itself, but by the behavioral changes the device enables. In clinical studies with medical devices for diabetes management, behavioral changes of study participants, not only those planned for the intervention group, but also unintended changes in control group participants, as well as those of the HCPs, should be taken into account and adequately considered in study design, implementation, and analysis.

Over the last several years, a number of studies have been published that investigated improvements in patient outcomes driven by interventions in their diabetes management with medical devices [2–16].

The Structured Testing Program (STeP) study and the two ProValue studies are examples of studies in which diabetes management interventions on clinical outcomes were investigated [17, 18]. All of these studies were multicenter cluster randomized controlled trials (RCTs) with the primary outcome being improvements in glucose control (HbA1c reduction) in patients with type 2 diabetes.

In the STeP study, subjects in the intervention group performed structured self-monitoring of blood glucose (SMBG) and received enhanced care, while subjects in the control group received standard of care. In the ProValue studies, two levels of ambulatory care, namely diabetes specialized practices (first ProValue study) and general medical practices (second ProValue study), were examined. The ProValue studies also had an identical study design, where subjects in the intervention group used an integrated Personalized Diabetes Management (iPDM) system to support their diabetes therapy while subjects in the control group were to receive standard of care. The primary outcome was an improvement in glycemic control of participants using the iPDM tools compared to the subjects in the control group.

The STeP and the ProValue studies reported significant differences in the reductions in HbA1c between the intervention and control groups; however, considerable improvements in glucose control were observed in the control groups as well [3, 19, 20]. Improvements in the control group reduced the size of the effect of the intervention in these studies. Similar outcomes were observed in other studies, which report conservative or weakened between-group differences due to improvements in the control group [6, 11, 14, 21]. Therefore, solely the recruitment to a clinical trial, independent of the interventions, resulted in improvements in HbA1c [22]. When designing an interventional study, such effects have to be taken into account; otherwise, the observed effects cannot be attributed to the defined intervention due to the profound effects of confounding factors. This is especially important when including behavioral interventions, as comprehensive support of HCPs is essential to utilize the full potential of the intervention.

This article aims to provide challenges in interpreting diabetes management intervention studies gained from the STeP and ProValue studies and suggests approaches for optimizing study implementation and for avoiding pitfalls for further studies with similar medical devices.

## Study implementation

### Engaging participants in the intervention group

A major difficulty in intervention studies that rely on behavioral changes is to ensure that the intervention triggers the desired effect (Table 1).

**Table 1:** Crucial aspects to consider in clinical studies with medical devices that rely on behavioral changes and options to reduce potential limitations including their challenges

Aspects to consider	Options to reduce limitations	Challenges
**Engaging participants in the intervention group**	**Optimal engagement of involved HCPs:** • Investigator meetings • Repeated training sessions of study sites • Peer-to-peer review	• Cost and time expensive • Consider optimal timing of training activities
	**Support in device handling** **on individual levels:** • Training for HCPs • Technical trainers should be familiar with study aims • Visualization of device-based intervention messages for subjects	• Cost and time expensive • Consider optimal timing of training activities
	**Detailed assessment of adherence:** • Questionnaires (e.g., about therapy adaptations, perceptions of tools) • Result analysis of both: “per protocol population” and “intention to treat population”	• Adherence to some instructions are difficult to trace
**Control group management**	**Cluster-randomization**	• Limited statistical power • Calculated cluster numbers have to be feasible, ethically justifiable, and affordable
	**Minimum attention to control group**	• Disease management is complex • Standard of care cannot be fully standardized • Standard of care is often less defined and monitored than the interventional treatment
	**Pilot trials**	• Cost and time expensive
	**Pretest periods**	• Cost and time expensive
	**Third study arm**	• Reduced statistical power ➔ requires more subjects ➔ cost and time expensive
	**Use of historical controls; retrospective data collection**	• Identification of a suitable control data • Consider progress in treatment standards
	**Using two separate protocols for the two groups**	• Informed consent forms should be well designed to avoid inclusion of interventional aspects
	**Two-stage randomization**	• Ethical concern
	**Adaptive study designs**	• Changes have to be planned and defined in advance • Implementation of adaptations has yet to be investigated

While in drug studies an intervention is defined by a given medication scheme that the participants simply have to follow, behavioral changes cannot be triggered that straightforward [23, 24].

There is no single intervention strategy to guarantee intervention adherence of all participants. Furthermore, a distinction between unintentional and intentional non-adherence should be taken into account. Whereas unintentional non-adherence depends on modifiable factors like poor understanding of the treatment or low health literacy or numeracy, in intentional non-adherence, participants rationally decide to not adhere by weighing the benefits and risk of following an intervention [25].

A set of key factors influencing attempts to improve participants’ unintentional non-adherence include a clear and effective communication between HCP and participant as well as realistic assessments of participants’ health literacy and knowledge. Health literacy is essential to the ability to adhere to study intervention as well as the ability to remember the details of the recommendations made to participants during visits influences the adherence. Thus, the explanation of the specific steps of the study regimen, review of the most important details, and written instructions may increase adherence. Furthermore, the risk of non-adherence is reduced with a more familiar relationship between HCP and participant. This enables HCPs to understand participants’ beliefs, attitudes, subjective norms, cultural context or social supports, and other relevant elements that are crucial for patient’s adherence [26, 27].

Many different behavior change techniques are already included in the standard of care in diabetes therapy, such as providing feedback on HbA1c levels and setting personal goals [25, 28].

In the ProValue studies, for example, the diabetes management intervention comprised a blood glucose meter and the corresponding software. Subjects in the intervention group had to perform structured SMBG with an individualized testing regimen selected by the treating physicians. This selection, as well as treatment adjustments, was supported by the diabetes management software. To ensure optimal engagement of the involved HCPs with a leading role in the implementation of the intervention, numerous local investigator meetings were organized. During these investigator meetings, HCPs of intervention group sites were trained by the coordinating investigator and the sponsor’s medical advisor in the use and concept of the diabetes management program. These meetings also enabled an exchange of experiences and individual patient cases could be discussed. HCPs could thus find a way to implement the iPDM into their daily practice and individual treatment patterns, and they were motivated to appropriately use the program. It was expected that this confidence and commitment of the HCPs would increase subjects’ compliance as well [29]. Inherently, the investigator meetings and the behavior changes of HCPs varied widely, partly because the initial knowledge and skills in the use of technology already differed between the study sites. Additional attempts for a standardization of handling iPDM would have increased comparability, but would have impaired the real-life aspects and competed with the need for actions individually tailored to each patient. As the desired behavioral changes are not expected to occur in one stroke but rather gradually in the course of the study and with the active use of the iPDM, repeated training sessions of study sites were supportive.

One instrument used in the STeP study was the “peer-to-peer review” that scheduled a review of intervention group patient data and subsequent therapy and advised by an uninvolved practitioner. The independent physician applied the same principles of the iPDM used in the regular study procedures and gave direct feedback to the treating physician on how to intensify or improve therapy adjustments. Even though the iPDM used in ProValue was constructed as an ongoing circle, most improvements were observed rapidly after study start [20]. This should be considered when the optimal timing of training activities has to be determined.

To be able to evaluate whether a behavioral intervention is successful, it is necessary to verify that recommendations have actually been followed. For the ProValue studies, this takes into account whether the HCP makes use of proposals made by the software and whether participants adhere to their HCP’s advices and instructions. While adherence to some instructions like the number of daily SMBG measurements can be assessed easily when device data are downloaded, other instructions like the consideration of SMBG results for insulin dosing decisions or dietary recommendations are difficult to be traced. Information on the intentional use of the intervention might be helpful in subgroup analysis to evaluate the effect of the intervention when completely implemented. In the ProValue studies, this was captured by questionnaires about the perception of the tools among HCPs and participants and by capturing detailed information about therapy adaptations and HCP recommendations. Data to identify and trace potentials and hurdles were available during each stage of the iPDM circle. In this regard, including an analysis of the “per protocol population” that fully adhered to the study intervention procedures is highly recommended in addition to results for the intention to treat population. Bartolo et al., for instance, could not show an advantage of a diabetes management intervention compared to standard care in their study [14]. However, they reported compliance to SMBG of less than approximately 50% in both groups and they reported larger improvements among patients that were more compliant. An inadequate use that did not lead to the intended behavior changes in the study might have been a reason for the limited effects.

**Fig. 1:**
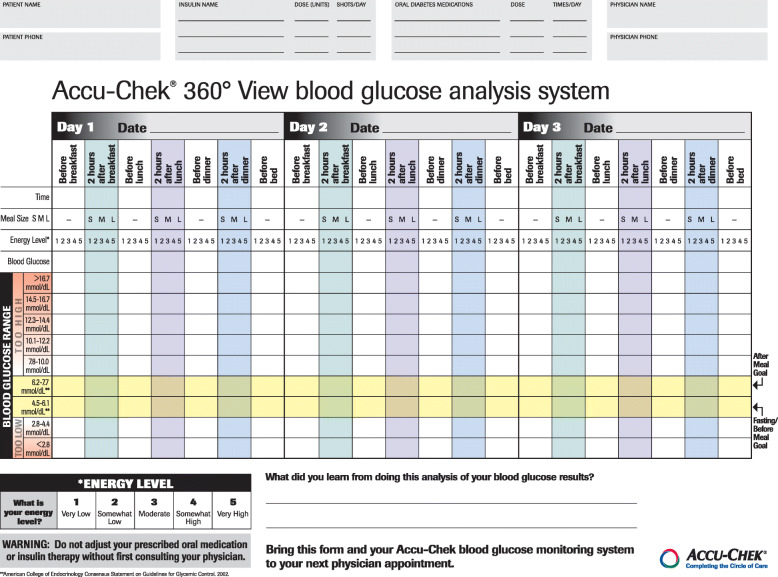
Paper tool with color grades for BG level documentation used in the STeP study

**Fig. 2:**
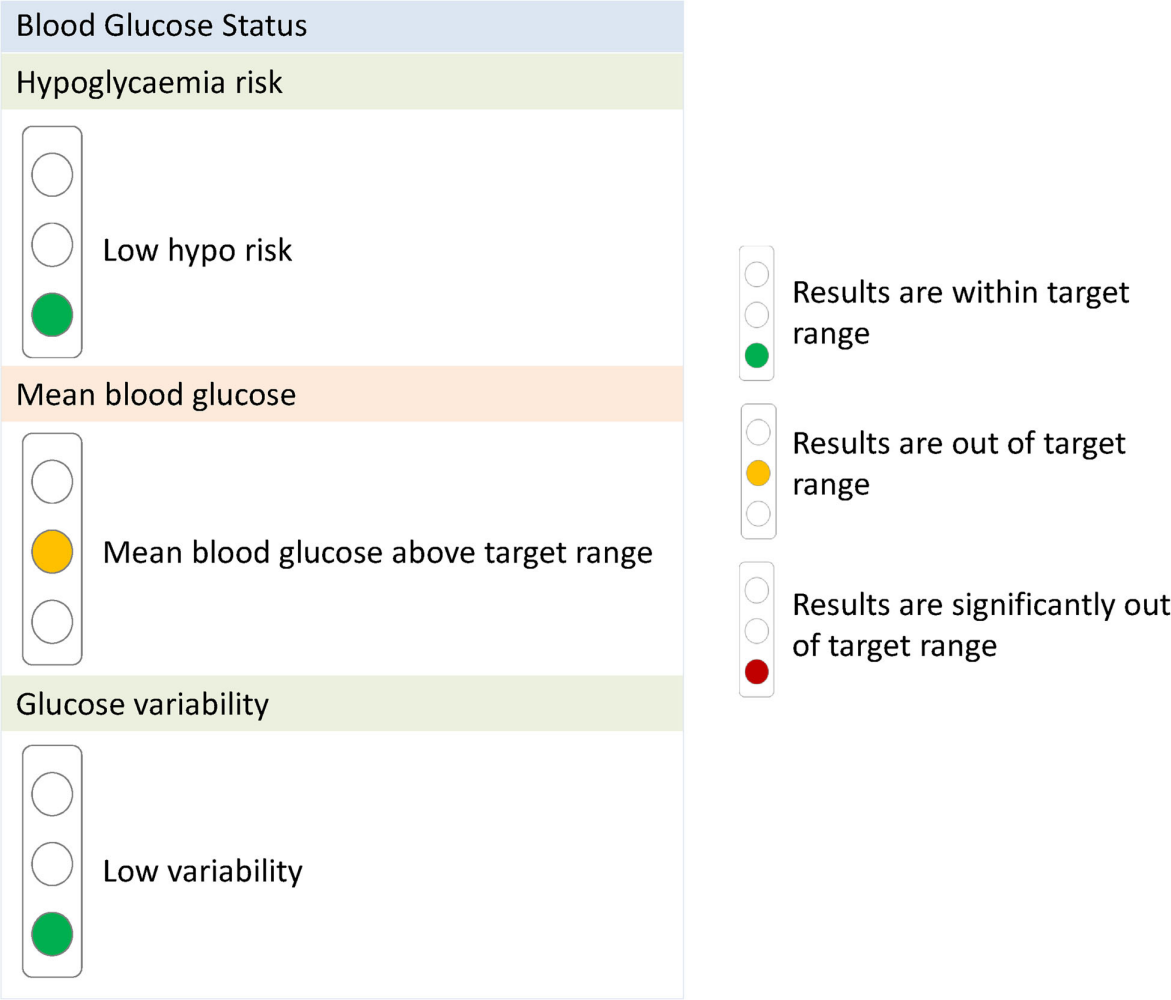
Traffic light scheme used in the ProValue studies

However, the introduction of a new medical device is time and energy consuming for HCPs. For example, software like the one used in the ProValue studies is, at least in Germany, not as common as one would expect at the present time, especially among general practitioners. Support in all technical issues should be provided on an individual level by study sponsors, adjusted to the particular knowledge and requirements, on demand and on site. Moreover, to be able to support the study sites in an appropriate and targeted manner, technical trainers have to be familiar with study aims and procedures too. In addition to technical training for the HCPs, messages from such a digital technology-based intervention have to be conveyed to the patients in a way they can understand. One lesson learned from STeP and ProValue is that visualization is an important factor. In STeP, patients graphically documented their blood glucose levels in a paper tool with color grades, while in ProValue, downloaded data were reported in a traffic light scheme (Figs. 1 and 2). Such visual feedback provides a link between technology, HCPs, and patients and facilitates the implementation of the intervention.

### Management of the control group

Another major difficulty in intervention studies that rely on behavioral change is to keep participants in the control group distant from the intervention, i.e., that behavioral changes desired in the intervention group do not occur in the control group as well (Table 1). While in drug studies finding an adequate control (e.g., a placebo) is mostly straightforward, control group design for behavioral studies is complex and the achievement of a truly “inactive” control group that strictly stays with standard care and does not change behavior is almost impossible. Usually, randomization and blinding are the preferred tools, but the implementation is not always feasible.

Adoption of behavioral changes requires an active involvement of both patients and HCPs; blinding is therefore not an option. If HCPs are also part of the intervention, like in STeP and ProValue studies, study personnel which gained knowledge from treating the intervention group subjects could transfer this to those in the control group, at least to a given extent. Thus, cluster-randomization, i.e., randomization of the study sites rather than the individual subjects, is necessary to avoid “contamination” of subjects in the control group [23]. This means cluster-randomization is suitable for interventions that are unlikely to be available for HCPs and patients in the trial [23].

Cluster-randomization, however, limits the possibility to control for differences between the sites, such as their implementation of standard of care. As the control sites are aware of the intervention done at the intervention sites, there is the risk that they tend to try to improve therapy in control group patients too and are more attentive to patient care than usual.

Statistical power of cluster-randomized studies is limited; they thus require larger sample sizes. Based on the variation between the clusters and the expected effect size, the optimal number of clusters, i.e., study sites, and subjects per cluster can be calculated [30]. However, scheduling the required numbers is often complicated by feasibility, ethical justifiability, and affordability, which may enforce the acceptance of compromises.

A limited effect size due to improvements also in the control group is an often-observed incident, caused by the so-called Hawthorne effect. This effect is attributable to subjects’ knowledge of being part of a study, i.e., being observed and having data collected. This study effect can improve the health status of a subject without any further intervention [31, 32]. Asking questions, for instance, induces rethinking about the current behavior and might induce respective changes [33]. Another reason for improvements in the control group is increased attention paid to the subjects by their HCPs. In principle, this increase in attention should be kept to a minimum; however, in reality, it is difficult to avoid. In addition, an intensive data gathering approach as used in the ProValue studies induces a high engagement of the participating HCPs (and also of the patients in both study groups) leading to improvements in the control group as well [20].

Also, the monitoring effort of clinical research associates (CRAs) regarding study implementation by HCPs, which is an absolutely necessary study procedure, has an impact on study implementation.

Limiting information about the intervention might be a possibility to reduce control group effect. This is not in strict compliance with the guideline for Good Clinical Practice. However, as long as the safety of study participants is paramount, a degree of concealment is accepted by research ethics committees for behavioral intervention studies [34]. According to this, all subjects and HCPs regardless of the study group have to be fully informed about the background and procedures of the study prior to the start. As such, in the ProValue studies, participants in the control group and HCPs were fully aware of the hypothesis that an iPDM and structured SMBG were expected to improve glycemic control. Participants randomized to the control group might therefore, whether or not intentionally, have sought for a comparable treatment or intensified their therapy on their own [31, 35]. Because of the detailed assessment of therapy adjustments and recommendations of HCPs in the ProValue studies, the main triggers for behavior changes that were identified for the subjects in the intervention group could also be detected among those in the control group [20]. Control group patients typically receive “standard of care” or “treatment as usual,” but these conditions are often less defined and monitored than the interventional treatment [29].

Standard care differs across countries, hospitals, and over time, depending on the respective health care provisions and updated guidelines and technologies that are introduced at variable rates. Especially in multicenter studies, the actual implementation might vary considerably between study sites and this cannot be controlled in cluster-randomized studies [36]. A clear definition of what is regarded as “standard” is essential for the validity of a study and should receive as much attention as the definition of the intervention. Mostly, patient care within a study is rather an enhanced standard of care for all the reasons discussed above. A meta-analysis of randomized control trials (RCTs) that investigated standard care conditions in control groups of behavior change studies in patients with diabetes showed that those control group patients that received a higher quality of standard care also showed larger improvements in study outcomes, thus reducing the effect size of the intervention [36].

Nevertheless, chronic disease management like diabetes therapy is complex and, like the intervention, standard of care cannot be fully standardized but has to be adapted to the individual patient and their compliance.

## Alternative study designs

Due to recruitment and “contamination” problems in interventional trials requiring behavioral changes, the realization of standard RCTs may be difficult [23]. Alternatives to the standard RCT when designing a medical device studies that rely on behavioral changes may be considered. While the above-described aspects concern the detailed implementation of a study, some variations in the general design might be considered with regard to the effect size, which was often observed to be lower than expected.

If control group effects are expected, it is essential to identify the particular reasons or triggers for behavior changes that may occur. Once identified, study procedures can be adjusted to avoid them or to even include them into the intervention. A pilot phase or study is indispensable to identify such factors and should therefore be included, especially if a large trial is planned. Therefore, more and more studies consider the additional effort of a pilot trial [7, 8, 11, 37, 38].

As the Hawthorne effect is described to be temporary and of relatively short duration [39], one approach towards a reduction of influencing the behavior of the subjects in the control group is to add one or several pretest periods to the study design [40, 41]. This means additional data collection before and after the pretest period, without an interventional treatment in any of the groups. Randomization and initiation of the intervention starts after this period using data obtained after the pretest as baseline data. Because it is expected that the majority of improvements induced by study effects occur between the first and the second data collection, the data used for the assessment of study outcomes will not be impaired, or at least less. However, the inclusion of a pretest period is cost and time expensive and might require a pilot study to determine an adequate duration. For STeP and the ProValue studies, a 3-month pretest period would have been sufficient, as the results indicate the strongest control group effects within the first 3 months of the study. Nevertheless, because these studies were accompanied by a lot of preparations for intervention group sites, such as training sessions, a postponement of randomization procedures would have interrupted the whole study flow.

One possibility for control conditions in RCTs is using a waitlist control [42]. Subjects of the control group that are on a waiting list, i.e., they expect to receive the active intervention at a later time point, have been shown to improve less than patients that receive only standard of care throughout the whole study [29]. A waiting control group could therefore be a more efficient way to influence the effect size than an inactive control group.

Other options include a third study arm to control for enhanced standard of care and study effects. Schwartz et al. proposed a design in which one arm receives the intervention, while the control condition has two arms, each with a crossover between a waiting list with standard of care and receiving the intervention [43]. Several data collection points are required for such a design. The crossover design reduces the heterogeneity within a group due to individually tailored implementation of the intervention, increasing statistical efficiency. Nevertheless, feasibility of a crossover depends on the kind of intervention and the expected long-term effects. Additionally, inclusion of further study arms reduces statistical power, and accordingly, it requires the inclusion of more subjects which also increases financial cost and study duration [41].

In this regard, retrospective data collection could be a feasible option, but only if required data are limited to standard assessments during usual patient visits, as expected when standard of care is claimed for control group subjects.

Use of historical controls, i.e., data assessed in other independent studies that already were conducted, is another promising option if study effects shall be reduced [44, 45]. In addition, with the use of historical controls, more resources become available for the intervention arm (which could be used for a larger sample size and therefore an increased power). Identification of a suitable control data set for the respective objective, however, is challenging, as well as the correct use of these data. In addition, the progress in treatment standards, assessment technologies, and other factors over time have to be considered.

A better separation of intervention and control group might be reached by using two separate protocols for the two groups. Consequently, all other participants such as CRAs should be exclusively assigned to one of the groups. The ProValue studies already worked with two protocols, but those were divided by the type of practice of the study sites rather than by study groups. A separate control group protocol would on the one hand enable a clear definition of “standard of care” and on the other hand allow a reduction of procedures in the control group to an absolute minimum. This applies not only to contacts between subjects and HCPs, but also between HCPs and further study staff. In addition, suitably designed informed consent forms should avoid inclusion of interventional aspects.

To prevent patients from consenting to therapy forms they may not get, a two-stage randomization could be another option. Accordingly, all patients give consent for follow-up first. An additional consent for study intervention is only provided to a randomly selected sample. Thus, patients randomized to the control group do not feel disadvantaged not receiving the intervention [46]. However, ethical concerns remain because there is only a personal consent to patient’s treatment and no full consent to the project from all patients [23, 46].

Adaptive study designs are becoming more and more common, however, not yet in medical device studies, but rather in drug studies, as adaptive designs are in particular effective in investigating dose-response relationships. Nevertheless, some of the several different approaches might also be used for medical device studies. Adaptive design means that procedures or conditions of a study are modified during the ongoing study based on results from interim analyses. However, these changes have to be planned and defined in advance [47, 48]. Adaptations include, e.g., randomization based on baseline data or sample size re-estimation to ensure the desired power. Implementation of adequate adaptations in studies including behavioral change has yet to be investigated. Nonetheless, such an approach could reduce the necessity of a separate pilot study and combine the exploratory and confirmatory stages of an investigation in one single study [38, 49]. Performance of an underpowered trial may furthermore be prevented [47]. In addition, it might be a better reflection of clinical practice if those patients that prove to be compliant and susceptible for an intervention are selected. Considerations about whether or not introducing new therapeutic options (might they be behavioral changes and/or diagnostic/treatment options) are usually made by HCPs based on their experiences with the respective patients.

## Conclusion

Based on experiences from the STeP and ProValue studies, several crucial aspects have to be considered in medical device studies when using interventions that rely on changes in behavior of study participants and their HCPs to achieve an effective implementation and significant results.

The article summarizes experiences gained from the three studies and provides suggestions for the implementation of other studies with similar medical devices.

In particular, definition of control group conditions and an integrative support of the intervention group have to be included. Improvements in the control group may reduce effect sizes and limit statistical significance; therefore, alternatives to the traditional RCT, like pretest periods or separate study protocols, are worth to be considered. As there is no ideal design for such studies, integration of experiences from other studies is essential to achieve the best possible study outcome.

## Data Availability

Not applicable

## Abbreviations

CGM: Continuous glucose monitoring
CRAs: Clinical research associates
HCPs: Health care professionals
iPDM: Integrated Personalized Diabetes Management
RCTs: Randomized controlled trials
SMBG: Self-monitoring of blood glucose
STeP: Structured Testing Program
